# Non-pharmacological Interventions for Improving Sleep Quality During Pregnancy: A Systematic Review and Meta-Analysis

**DOI:** 10.1055/s-0042-1746200

**Published:** 2022-05-23

**Authors:** Daiane Sofia Morais Paulino, Carolina Bicudo Borrelli, Débora Bicudo Faria-Schützer, Luiz Gustavo Oliveira Brito, Fernanda Garanhani Surita

**Affiliations:** 1 Department of Obstetrics and Gynecology, Universidade Estadual de Campinas, Campinas, SP, Brazil

**Keywords:** sleep quality, non-pharmacological interventions, pregnant women, systematic review, meta-analysis, qualidade do sono, intervenções não-farmacológicas, gestantes, revisão sistemática, metanálise

## Abstract

**Objective** To investigate the effect of non-pharmacological interventions to improve sleep quality during pregnancy.

**Data sources** A search was made in the NCBI/PubMed, ClinicalTrials.gov, Embase, BVS, and Web of Science databases. There were no limitations regarding language, sample size, and type of non-pharmacological intervention. We have included prospective clinical trials between July 2014 and July 2019.

**Selection of studies** This study was registered in the Prospective International Registration of Systematic Reviews (PROSPERO) database was performed. Publication bias was also assessed with funnel plots. the primary outcome was the total score in the Pittsburgh Sleep Quality Index (PSQI) before and after intervention. Risk of bias and the Grading of Recommendations Assessment, Development, and Evaluation (GRADE) criteria were used for assessing methodological quality. From the 28 retrieved studies, we have selected 8 for qualitative analysis and 6 for meta-analysis.

**Data collection** Two independent reviewers performed the study selection. In the case of disagreement, a third senior reviewer was consulted. The study was initially assessed based on the title, followed by abstract. Lastly, the full text was assessed to be included.

**Data Synthesis** A significant improvement on the sleep quality (PSQI score) was observed when all interventions were grouped (MD = -3.03, 95%CI -4.15 to -1.92, *n* = 623, i ^2^ = 84%, *p* < 0.001). Analysis by subgroup (music listening: MD = -1.96, 95% CI -3.27 to -0.65, *n* = 207, i ^2^ = 67%, *p* = 0.003 and other interventions: MD = -3.66, 95% CI -4.93 to -2.40, *n* = 416, i2 = 80%, *p* < 0.001) showed an improvement, with high heterogeneity. Risk of bias has shown performance and detection bias for almost studies, and GRADE evidence was very low for all analyzed variables.

**Conclusion** Non-pharmacological interventions—listening to music, physical exercise, relaxation exercises, lettuce seed, sleep hygiene, and acupressure—are effective for improving sleep quality during pregnancy.

## Resumo

**Objetivo** Investigar o efeito de intervenções não-farmacológicas para melhorar a qualidade do sono durante a gravidez.

**Fontes dos dados** Uma busca foi feita nas bases de dados NBCI/PubMed, ClinicalTrials.gov, Embase, BVS, e Web of Science. Não houve limitações quanto ao idioma, tamanho da amostra e tipo de intervenção não-farmacológica. Incluímos estudos clínicos prospectivos entre julho de 2014 e julho de 2019.

**Seleção dos estudos** Foi realizado um registro no banco de dados *Prospective International Registration of Systematic Reviews* (PROSPERO). O viés de publicação foi avaliado com gráficos de funil. O desfecho primário foi a pontuação total do *Pittsburgh Sleep Quality Index* (PSQI) antes e depois da intervenção. O risco de viés e os critérios *Grading of Recommendations Assessment, Development, and Evaluation* (GRADE) foram usados para avaliar a qualidade metodológica. Dos 28 estudos encontrados, selecionamos 8 para análise qualitativa e 6 para a metanálise.

**Coleta de dados** Dois revisores independentes realizaram a seleção dos estudos. Em caso de discordância, um terceiro revisor foi consultado. Inicialmente o estudo foi avaliado com base no título e resumo. Para a inclusão, foi avaliado o texto completo.

**Síntese dos dados** Uma melhora significativa na qualidade do sono (PSQI score) foi observada quando todas as intervenções foram agrupadas (MD = -3.03, 95% CI -4.15 a -1.92, *n* = 623, i ^2^ = 84%, *p* < 0.001). A análise por subgrupo (escutar música: MD = -1.96, 95% CI -3.27 a -0.65, *n* = 207, i ^2^ = 67%, *p* = 0.003 e outras intervenções: MD = -3.66, 95% CI -4.93 a -2.40, *n* = 416, i2 = 80%, *p* < 0.001) também mostrou uma melhora da qualidade do sono, porém com alta heterogeneidade. A análise do risco de viés mostrou que quase todos os estudos avaliados apresentaram viés de desempenho e detecção, e o nível de evidência GRADE foi muito baixo para todas as variáveis analisadas.

**Conclusão** Intervenções não-farmacológicas - ouvir música, exercícios físicos, exercícios de relaxamento, sementes de alface, higiene do sono e acupressão - são eficazes para melhorar a qualidade do sono durante a gravidez.

## Introduction

Sleep quality and sleep routine are significantly affected by the hormonal, physical, and psychological changes that occur during pregnancy. It has been described that increased progesterone and estrogen levels, as well the discomfort caused by fetal growth and fetal movements, lumbar pain, gastroesophageal reflux, nocturnal cramps, frequent urination, and concerns inherent to the baby's health have a negative influence on subjective sleep quality. 1 2

Studies have shown that poor sleep quality in pregnant women varies from 39.6 to 89.3%, and it is observed that sleep disorders seem to worsen throughout pregnancy. 3 4 5 Poor sleep quality during pregnancy impacts on labor, and maternal and fetal heath. It has been demonstrated that the quality of sleep during late pregnancy may predict the length of labor and mode of labor. 6 7 Low birthweight and Apgar scores are significantly correlated with duration of sleep. 8 Furthermore, poor sleep quality impacts on the woman's quality of life 9 and increases the risk of gestational diabetes, hypertensive disorders, and postpartum depression. 10 11 12 13

Some sleep medications, such as flurazepam, temazepam, and mefloquine, have their use contraindicated for having teratogenic effects. 14 Other drugs, like zolpidem, are not teratogenic but may cause adverse maternal-fetal outcomes such as preterm delivery. 15 In addition, pregnant women are generally cautious about the use of drugs during pregnancy. Thus, given the health-related consequences of sleep disorders and the difficulty of using sleep-improvement drugs during pregnancy, there has been a growing interest in non-pharmacological interventions to improve sleep quality during pregnancy.

Here, we aim to investigate the effect of non-pharmacological methods for improving sleep quality in pregnancy by a systematic review and meta-analysis.

## Methods

### Protocol and Registration

A systematic review was conducted, and it was registered in the Prospective International Registration of Systematic Reviews (PROSPERO) () database with register number CRD42018092004.

### Eligibility criteria

The inclusion criteria were defined using the P (participants) I (intervention) C (comparison) O (outcome) S (study design) strategy. We have included pregnant women that were submitted to non-pharmacological interventions (with a control group without intervention or with the usual care) during pregnancy for sleep improvement (via the Pittsburgh Sleep Quality Index [PSQI] or other methods as the mean outcome) in prospective clinical trials (CTs) between July 2014 and July 2019. There were no limitations regarding language, study sample size, and type of non-pharmacological intervention.

### Search Strategy and Study Selection

The following databases were consulted: NCBI/PubMed, ClinicalTrials.gov, Embase, Biblioteca Virtual em Saúde- BVS and Web of Science, and the following medical scientific terms (Medical Subject Headings - MeSH) with their synonyms were considered for the development of the search strategy: *Sleep Quality* , Pregnant women. The following search strategy was developed on the PubMed database and modified according to each database requirement: ([ *sleep quality* ) AND (Pregnant Women [MeSH Terms]) OR *Pregnant Women* (Title/Abstract) OR *Women* , *Pregnant* (MeSH Terms) OR *Women* , *Pregnant* (Title/Abstract) OR *Pregnant* *Woman* (MeSH Terms) OR *Pregnant* *Woman* (Title/Abstract) OR Woman, Pregnant (MeSH Terms) OR *Woman* , *Pregnant* [Title/Abstract]).

Two independent reviewers (D. S. M. P. and C. B.) performed the study selection and, in the case of disagreement on the final inclusion of a study, a third senior reviewer (F. G. S.) was consulted. The study was initially assessed based on the title, followed by abstract. Lastly, the full text was assessed to be included. The references listed in the selected articles were also consulted, aiming to find any other study that had not been previously identified by the search strategy. Study authors were contacted whenever necessary for further clarification or information regarding their articles.

### Outcomes

The data of sleep quality assessed by the PSQI total score, as well as the number of participants were extracted from each study included in the meta-analysis. The PSQI was developed in 1989 by Buysse et al. 16 to assess sleep quality during the last four weeks of pregnancy. The PSQI is a self-report questionnaire with 19 items, which measures 7 components of sleep quality: subjective sleep quality, sleep latency (time necessary to fall asleep and frequency of not falling asleep in 30 minutes), sleep duration, habitual sleep efficiency, sleep disturbances, use of sleep medication and daytime dysfunction. The components are scored between 0 and 3, and the total score is obtained by the sum of the 7 components with a value range of 0 to 21. Total score > 5 is clinically classified as poor sleep quality.

Two reviewers (D. S. M. P. and C. B.) independently assessed the risk of bias in the included studies using the Cochrane risk-of-bias assessment tool. 17 Disagreements were resolved by consultation with a third reviewer (L. G. B.), and consensus was obtained through discussion. About the overall quality of the body of evidence for the review outcomes, we used the Easy Grade Pro software (Orbis Technologies, Inc., Annapolis, MD, USA). The Grading of Recommendations, Assessment, Development and Evaluation (GRADE) criteria consider study limitations, consistency of effect, imprecision, indirectness, and publication bias.

### Statistical Analysis

The meta-analysis was performed for at least two studies that could be grouped into a single forest plot. For continuous outcomes, the mean difference with 95% confidence intervals was calculated. We stipulated a 5% significance level. Heterogeneity was calculated by the I ^2^ test, and if values were over 50%, a random-effect model was performed. The outcomes were divided according to the type of intervention (music intervention and other interventions) for subgroup analysis to reduce heterogeneity. The data were tabulated and analyzed using the RevMan 5.3 software (The Cochrane Collaboration, Copenhagen, Denmark).

A forest plot was performed to evaluate the relative strength of the intervention effect. Funnel plots were built to assess publication bias. For database validation, the first author has inserted the data, and a second author (C. B.) revised the database to identify possible inconsistencies or typos.

## Results

**Fig. 1:**
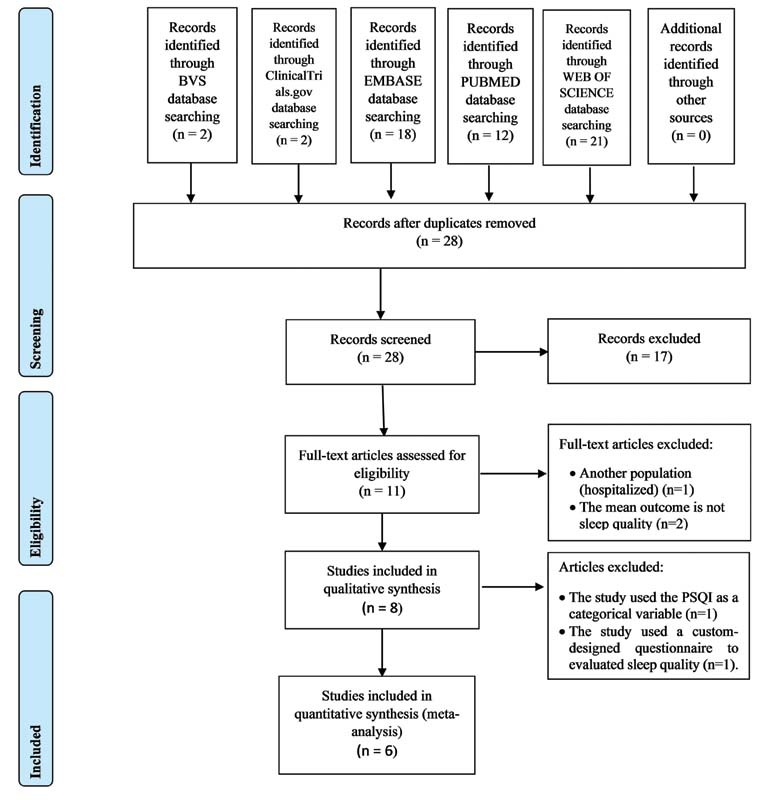
Preferred reporting items for systematic reviews and meta-analyses (PRISMA) flowchart of the study screen process.

A total of eight studies were retrieved after study selection; six of them were suitable for meta-analysis. Search history and screen process of the articles were detailed in the flow diagram presented in Figure 1 .

All studies included in the meta-analysis were randomized clinical trials and used the PSQI total score to measure sleep quality. The other two studies included in qualitative review were non-randomized clinical trials, 18 19 one of which used the PSQI as a categorical variable (with score ranging from 7 to 21 labeled as moderate-to-severe insomnia), and the other used a custom-designed questionnaire to evaluate sleep quality. 19 Studies were performed in six countries. Sampling for these studies ranged from 42 to 134 in the intervention group, and 42 to 101 in the control group, comprising 1,020 pregnant women. Non-pharmacological interventions studied to improve sleep quality were listening to music ( *n* = 2), 20 21 physical exercise ( *n* = 2), 19 22 relaxation exercises, 23 lettuce seed, 24 sleep hygiene, 25 or acupressure. 18 Treatment duration varied across the studies. Other details of the studies included in the review were described in Table 1 .

**Table 1:** Summary of clinical trials that assessed the effect of non-pharmacological strategies for improving sleep quality

Study ID	Country	Year	Sample size, N		Participant criteria	Intervention		Instrument
			Intervention	Control		Strategies/Intervention	Duration	
Özkan and Rathfisch 23	Turkey	2018	42	42	3rd trimester, ≥ 20 y, primiparous, singleton, HRP, GA 28–34 weeks, pre-pregnancy BMI ≤ 25 kg/m ^2^ , neck circumference < 38 cm, and no RLS.	Listening to a relaxation exercises CD before sleeping. The CD comprised a 4-minute introduction, 10-minute of information on deep relaxation PE and points to consider during PE, 30-minute introduction to relaxation, and the last 30 minute includes relaxation music.	4 weeks	PSQI
Pour et al. 24	Iran	2018	50	50	20–45 y, singleton pregnancy, GA 12–36 weeks, with insomnia and PSQI score > 5.	IG received capsules containing 1000 mg of lettuce seed daily. CG received placebo capsules containing starch.	2 weeks	PSQI
Rodriguez-Blanque et al. 22	Spain	2018	67	67	GA 12–20 weeks, without any absolute contraindications for PE * .	IG took part in the SWEP program, performing three 1-hour sessions/week water exercises. CG followed the usual recommendations, including emphasis on the positive effects of PE.	17 weeks	PSQI
Sönmez and Derya 25	Turkey	2018	64	64	Diagnoses of RLS, literate, at 3rd trimester, HRP, using iron supplementation, without sleep disorder.	IG received sleep hygiene training. A sleep hygiene training booklet was issued after training, and 2 weeks later, during a home visit, participants received a counseling service on sleep hygiene.	4 weeks	PSQI
Kocsis et al. 19	Romania	2017	79	53	HRP, 18–40 y, GA 18–22 weeks, BMI < 35 kg/m ^2^ , parity < 3.	IG followed a specific PE program under strict instruction by a PE training specialist. The PE program structure involved 2-hour training sessions twice a week. PE included posture correction, preserving muscle tone, and strengthening pelvic and posterior muscles, breathing exercises, and relaxation techniques.	10 weeks	CDQ **
Neri et al. 18	Italy	2016	134	101	HRP, singleton pregnancy, ability to understand Italian, with feelings of anxiety and poor sleep quality.	IG was advised by a midwife (trained by an expert acupuncturist) to wear a soft rubber pin kept in place by an adhesive plaque able to exert acupressure on Point 7 of the heart meridian.	2 weeks	PSQI
Shobeiri et al. 20	Iran	2016	42	44	PSQI score > 5, 18–35 y, GA 30–34 weeks, singleton pregnancy, no drug addiction, not taking drugs affecting sleep quality, avoiding antidepressants use, without mental/physical disorders, access to an audio player at home.	IG received music therapy counseling in two weekly sessions, with each session lasting 60 minutes, in groups of 5–7 people. The music therapy method was passive music-listening (instrumental music by Kitaro, a Japanese composer and performer).	4 weeks	PSQI
Liu et al. 21	Taiwan	2016	61	60	PSQI score > 5, > 18y, GA 18–34 weeks.	IG was instructed to listen to at least one disc (30-minute) of the five prerecorded CDs compiled by the researcher or a minimum 30 minute of their preferred music per day at bedtime for 2 weeks. CG received the usual prenatal care.	2 weeks	Chinese version of the PSQI

Abbreviations: BMI, body mass index; CD, compact disk; CDQ, custom-designed questionnaire; CG, control group; GA, gestational age; HRP, habitual risk pregnancy; IG, intervention group; PE, physical exercise; PSQI, Pittsburgh Sleep Quality Index; RLS, restless legs syndrome; SWEP, study of water exercise in pregnancy.

* Described by the American College of Obstetricians and Gynecologists.

** Involved general perception of sleep quality and quantity, number of awakenings, difficulty falling asleep, insomnia, restless sleep, snoring, diurnal sleep, and consequences of inadequate sleep.

**Fig. 2:**
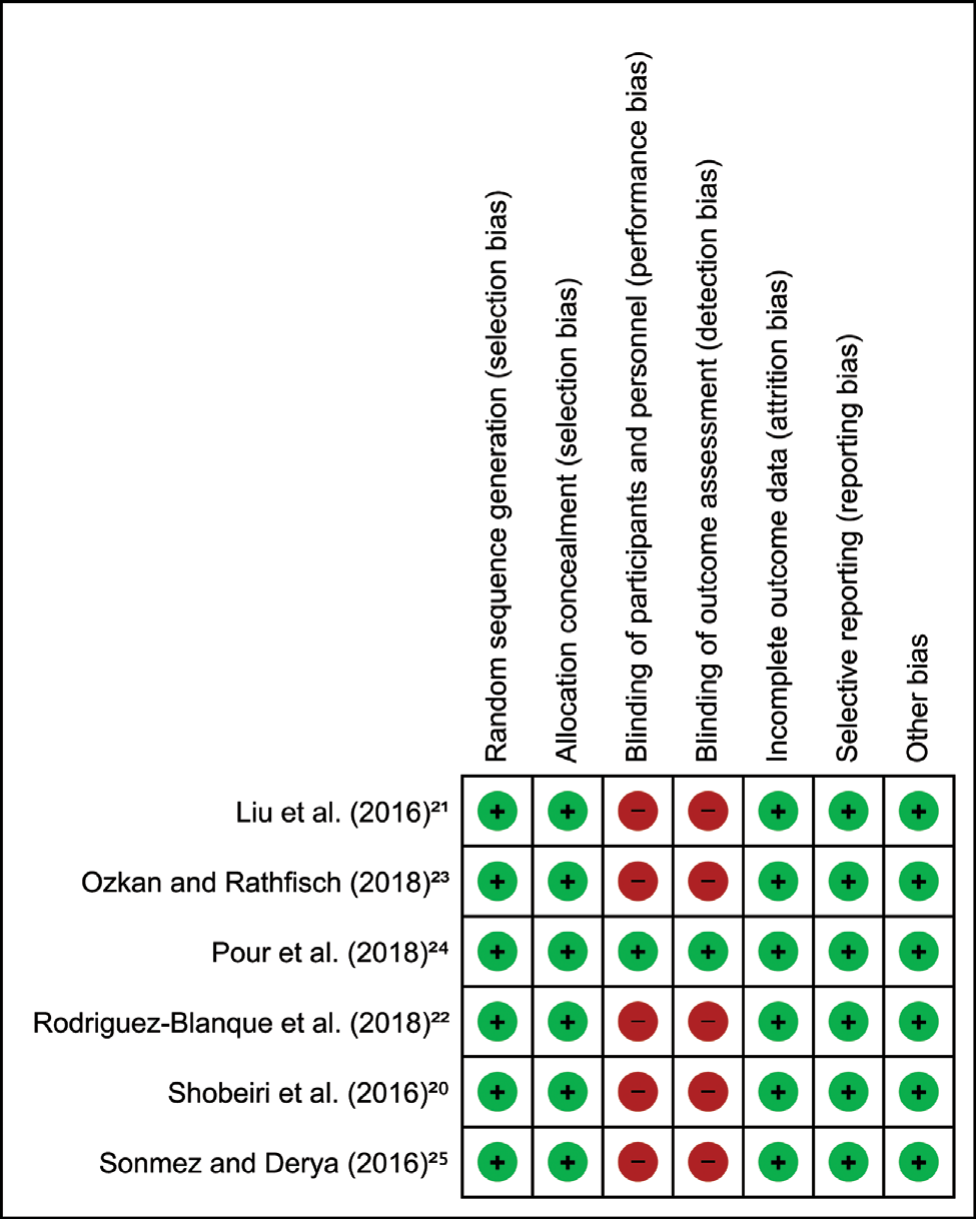
Risk of bias summary: review authors' judgements about each risk of bias domain. Based on the Cochrane risk-of-bias assessment tool. Green color indicates low risk of bias; red color means indicates risk of bias.

Figure 2 illustrates the risk of bias assessment of the included studies. All studies in the meta-analysis described the random sequence generation method and allocation concealment. Given the type of interventions used, only one study has blinded the participants, 24 and almost all studies were classified as high risk of bias. Similarly, most of the studies did not blind the investigators. We classified the studies as having a low risk of bias for incomplete outcome when they reported the adherence and/or dropout rates; and if they properly use methods to control the attrition, and if they did not have inappropriate methods for imputing missing data. In relation to selective reporting, we scored all studies as low risk of bias, because the trials selected reported all predetermined outcomes. Other bias sources were not identified in the trials.

We observed a very low evidence quality and the main limitations of the included studies were also evaluated according to GRADE criteria. The details regarding quality of evidence can be found in Table 2 . Indirect evidence was not serious at the trial that studied music listening, but very serious in the other interventions.

**Table 2:** Grading of Recommendations Assessment, Development, and Evaluation criteria for Pittsburgh Sleep Quality Index score among the eligible randomized controlled trials

Certainty assessment						Participants, n (before and after)		Absolute effect (95% CI)	Certainty
Number of studies	Study design	Risk of bias	Inconsistence	Indirect evidence	Imprecision	IC	CG		
**Non-pharmacological interventions - Music listening**									
2	Randomized clinical trial	Severe ^a^	Severe ^b^	Not severe	Severe ^c^	103	104	MD − **1.96** (−3.27 to −0.65)	⊕⚪⚪⚪ Very low
**Non-pharmacological interventions - Other interventions**									
4	Randomized clinical trial	Severe ^a^	Severe ^b^	Too severe ^d^	Severe ^c^	208	208	MD − **3.66** (−4.93 to −2.4)	⊕⚪⚪⚪ Very low

Abbreviations: CI, confidence interval; CG, control Group; IC, intervention group; MD, mean difference.

Explanations: a. Lack of blinding was present in all RCTs; b. Heterogeneity varied from 67–80% even after using random-effect models; c. Sample size for each group in total is less than 400 individuals; d. Indirect evidence due to different populations (one study is from patients with restless leg syndrome) and this subgroup analysis also present different interventions (four in total).

**Fig. 3:**
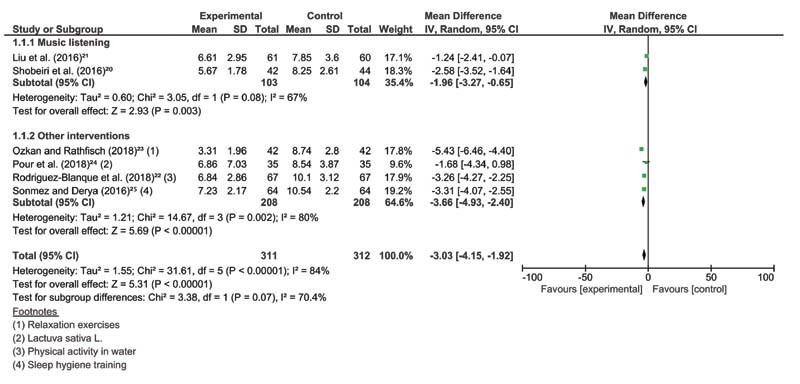
Forest plot of all non-pharmacological interventions and their subgroups, outcome: sleep quality (measured by PSQI total score).

From 28 retrieved studies, we have selected 8 for qualitative analysis and 6 for meta-analysis ( Fig. 3 ), comprising 1,020 pregnant women.

**Fig. 4:**
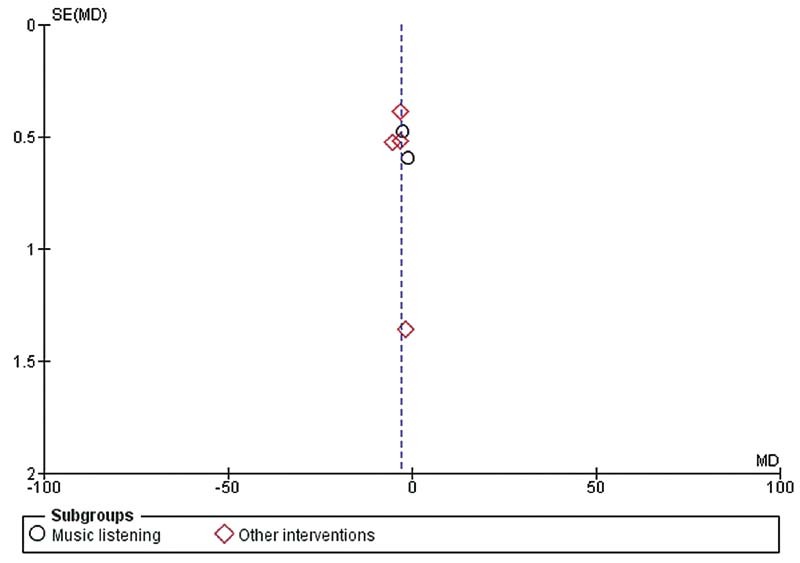
Funnel plot estimating the risk of publication bias.

A significant improvement on the sleep quality (PSQI score) was observed when all interventions were grouped (mean difference [MD] = -3.03, 95% confidence interval [CI] -4.15 to -1.92, *n* = 623, i 2 = 84%, *p* < 0.001). We also performed two subgroups of analyses based on the interventions: music listening and other interventions. Analysis by subgroup (music listening: MD = -1.96, 95% CI -3.27 to -0.65, *n* = 207, i 2 = 67%, *p* = 0.003 and other interventions: MD = -3.66, 95% CI -4.93 to -2.40, *n* = 416, i 2 = 80%, *p* < 0.001) showed an improvement on sleep quality, with high heterogeneity. The risk of bias has shown good performance, however with some bias present in the majority of studies, GRADE evidence was low for all analyzed variables. The assessment of publication bias was demonstrated in the funnel plot ( Fig. 4 ), which suggests absence of publication bias, given the symmetry displayed.

## Discussion

Our systematic review has demonstrated that non-pharmacological interventions improved sleep quality during pregnancy. Several studies have demonstrated that women during pregnancy experienced a poor sleep quality, and this may be a target of intervention given the disturbers of sleep can predict adverse maternal and fetal outcomes; 26 27 28 29 30 31 however, to our knowledge, this is the first review to evaluate the effects of lifestyle interventions for promoting sleep quality improvement during pregnancy. We have also calculated the risk of bias of these studies and the quality of evidence using GRADE, giving an idea about the level of information provided in these studies.

The PSQI total score was the instrument assessed as a measure of sleep quality in our findings. Many studies in this field are based on self-reported measures of sleep quality, and although there is variation in the agreement between subjective and objective sleep measures, 6 32 33 the use of subjective parameters is an accurate predictor of complications in pregnancy and the postpartum period. 34 35 36

Given the importance of methodological quality in the validity of computed tomography (CT) results, it is crucial to evaluate the risk of bias. Here, five of the six studies included in the quantitative analysis had high risk of bias in at least one domain, predominantly related to lack of blinding. However, given the nature of the interventions, most of the studies could not blind the participants. Our assessment also found that risk of bias among investigators could have been avoided if an investigator blinded to the group allocations had been recruited for data collection. However, as PSQI is a structured questionnaire, possibly minimal bias was introduced by the lack of a blinded investigator.

Our systematic review included studies from six different countries, but from two regions (Europe and Middle East), limiting the representativeness of our sample. In this sense, we need to be cautious in extrapolating these data to the general population. In addition, future higher-quality studies should provide a detailed of non-pharmacological measures for sleep improvement in other populations.

One study in our meta-analysis was conducted on women with diagnoses of restless legs syndrome (RLS). This condition is well explored during pregnancy and has been demonstrated to be more frequent during pregnancy than in the general population, 37 RLS has also been associated with decreased sleep quality. 38 39 40

A recent meta-analysis of sleep quality during pregnancy 28 has indicated a mean PSQI score of 6.07 and a 1.68-point increase in the mean PSQI score from the second to third trimester. In our findings, there was a decrease of 3.03 points in the mean difference of PSQI total score, suggesting that lifestyle interventions during pregnancy may be an important way to minimize the sleep disturbances of pregnant women.

The subgroup analysis showed that listening to music was effective in improving sleep quality in pregnancy. However, it is worth noting that there is a low level of scientific evidence in this statement.

Interestingly, despite the benefits of non-pharmacological measures demonstrated in the current meta-analysis, this approach is still little used in clinical practice.

This study has some limitations that should be considered. First, the low number of well-controlled trials using non-pharmacological interventions for sleep problems in pregnant women that are available to be included in a meta-analysis. Moreover, we could not extract any quantitative data from two eligible studies. 18 19 The main reasons were that one study 18 used the PSQI as a categorical variable (% of women with a PSQI score ranging 7–21), and although the authors answered our e-mail, they did not provide any additional data. The other study used a custom-designed questionnaire to evaluate sleep quality 19 ; as a result, the parameters that assessed sleep quality are different from those of the PSQI. The eligible studies had considerable differences between the intervention type, time and duration, and the population studied. Consequently, our results should be analyzed carefully. Moreover, the sample size for each group was < 400 participants, and the heterogeneity was 67 to 84%, even after using random-effect models, rendering the strength of recommendation weak. As the number of studies were not sufficient to perform a meta-regression or sensitivity analysis, we need to be cautious about the generalizability of data. Despite these limitations, our findings identify strategies that may be adopted in prenatal care and should be considered by health professionals aiming to improve sleep patterns during pregnancy and also for avoiding possible adverse effects related to sleep disturbances, not only in pregnancy but also postpartum.

## Conclusion

Safe strategies for improving sleep quality in pregnant women should be emphasized during prenatal care. The current analysis shows that non-pharmacological interventions (listening to music, physical exercise, relaxation exercises, lettuce seed, sleep hygiene, and acupressure) are effective for improving sleep quality during pregnancy. Although the evidence quality was very low. Moreover, lifestyle interventions and the encouragement of health behaviors during the gestational period may contribute to avoiding adverse outcomes for mother and fetus. Furthermore, considering the high prevalence of poor sleep in pregnant women and the limitations in drug use during the gestational period, future investigations that consider lifestyle modifications for improving sleep in other populations should be performed. Finally, further studies examining the potential effect of non-pharmacological approaches in postpartum women should be conducted, once the prevalence of poor sleep quality is also increased during the postpartum period.
